# Fermentation Results and Chemical Composition of Agricultural Distillates Obtained from Rye and Barley Grains and the Corresponding Malts as a Source of Amylolytic Enzymes and Starch

**DOI:** 10.3390/molecules21101320

**Published:** 2016-10-01

**Authors:** Maria Balcerek, Katarzyna Pielech-Przybylska, Urszula Dziekońska-Kubczak, Piotr Patelski, Ewelina Strąk

**Affiliations:** Department of Spirit and Yeast Technology, Institute of Fermentation Technology and Microbiology, Faculty of Biotechnology and Food Sciences, Lodz University of Technology, Wolczanska 171/173, Lodz 90-924, Poland; katarzyna.pielech-przybylska@p.lodz.pl (K.P.-P.); urszula.dziekonska-kubczak@p.lodz.pl (U.D.-K.); piotr.patelski@p.lodz.pl (P.P.); ewelina.strak@dokt.p.lodz.pl (E.S.)

**Keywords:** rye, barley, cereal malts, alcoholic fermentation, natural products, agricultural distillate, spirit beverages

## Abstract

The objective of this study was to determine the efficiency of rye and barley starch hydrolysis in mashing processes using cereal malts as a source of amylolytic enzymes and starch, and to establish the volatile profile of the obtained agricultural distillates. In addition, the effects of the pretreatment method of unmalted cereal grains on the physicochemical composition of the prepared mashes, fermentation results, and the composition of the obtained distillates were investigated. The raw materials used were unmalted rye and barley grains, as well as the corresponding malts. All experiments were first performed on a semi-technical scale, and then verified under industrial conditions in a Polish distillery. The fermentable sugars present in sweet mashes mostly consisted of maltose, followed by glucose and maltotriose. Pressure-thermal treatment of unmalted cereals, and especially rye grains, resulted in higher ethanol content in mashes in comparison with samples subjected to pressureless liberation of starch. All agricultural distillates originating from mashes containing rye and barley grains and the corresponding malts were characterized by low concentrations of undesirable compounds, such as acetaldehyde and methanol. The distillates obtained under industrial conditions contained lower concentrations of higher alcohols (apart from 1-propanol) than those obtained on a semi-technical scale.

## 1. Introduction

The spirit drinks sector is important for consumers, producers, and the agricultural industry in the European Union. The raw materials used by this sector in European countries include agricultural products, such as cereal grains, potatoes, sugar cane, sugar beets, and wine. It is noteworthy that 85% of the output is produced from grains [1].

Spirit beverages made from fermented grain mashes are popular and highly respected around the world for their flavor and taste [2]. Rye (*Secale cereale*) is a cereal crop that has been cultivated in Europe since ancient times [3] and Poland has a long tradition of making Starka from aged raw rye spirit. The raw material for the production of Starka is rye distillate (raw spirit) of suitable organoleptic properties and an alcoholic strength by volume (ASV) of 91% to 92% [4].

Polish vodka is among the spirits known and respected around the world. The geographical indication “Polish Vodka” has been registered in Annex III to the Regulation (EC) No. 110/2008 of the European Parliament and of the Council [5]. According to Polish legislation [6], the name “Polish Vodka” can only be applied to vodka originating from agricultural ethanol obtained exclusively from traditional cereals (rye, wheat, barley, oats, and triticale) and potatoes grown in Poland and also the entire process of its production and bottling must be carried out in this country.

Besides grain spirits, cereal malts (and in particular barley malt) are used in the production of many distilled beverages because of their flavor enhancement properties [7]. Malt-based spirit beverages known as whiskies are made in a number of countries, with the major producers being Scotland, Ireland, and Japan. Each country has a different definition of what constitutes malt whisky, with corresponding legislation. In Scotland and Ireland, the legal definitions are very specific. Scotch malt whisky is made exclusively from malted barley with no other grains included [8].

Scotch whisky has two major and distinct forms, malt whisky and grain whisky. Malt whisky is produced entirely from barley malt, while Scotch grain whisky is made from unmalted cereals processed together with a small amount of barley malt, which supplies the enzymes converting starch to fermentable sugars. The production of Scotch grain whisky, similarly to that of malt whisky, is regulated by the provisions of the 1988 Scotch Whisky Act and the 1990 Scotch Whisky Order (Statutory Instrument 1990), which were enacted to protect the exclusive generic nature of Scotch whisky from adulteration by other spirits. According to this legal definition of Scotch whisky, only endogenous enzymes from the malt can be used for starch conversion and additional commercial enzyme preparations are not permitted [9].

The rising requirements on foods and alcoholic beverages (especially on organic products) are factors indicating that technological innovations should be applied both to improve the quality of traditional spirits and to create new, original spirit drinks.

Cereal malt is a particularly suitable source of enzymes and starch in the production of organic spirit distillates, taking into account the fact that the commonly used commercial enzyme preparations of microbial origin are often derived from genetically modified organisms, contravening the principles of organic production [10].

The objective of this study was to determine the efficiency of rye and barley starch hydrolysis in the mashing processes using cereal malts as a source of amylolytic enzymes and starch. In addition, the effect of the malts on the physicochemical composition of the obtained mashes, fermentation results, and the composition of the resulting distillates was investigated.

## 2. Results

### 2.1. The Chemical Composition of Raw Materials

Analysis of the tested raw materials (see Table 1) showed moisture content in grain at (88.0 ± 6.0) g/kg for unmalted rye and (87.0 ± 5.0) g/kg for unmalted barley, while in cereal malts it was (39.0 ± 2.0) g/kg for barley and (42.0 ± 2.0) g/kg for rye.

Starch content amounted to (621.0 ± 15.2) g/kg for rye grain cv. Dańkowskie Diament and (485.0 ± 14.5) g/kg for barley grain cv. Karakan. In cereal malts, starch content was lower and ranged from (382.6 ± 17.65) g/kg for barley to (410.0 ± 16.53) g/kg for rye.

The obtained results show a significant difference between the amount of reducing sugars contained in unmalted cereals ((15.2 ± 1.3) g/kg for rye and (19.3 ± 1.0) g/kg for barley) and in cereal malts (166.2 ± 12.5 g/kg for barley malt and 186.5 ± 15.5 g/kg for rye malt).

As regards protein content, unmalted rye and barley grains contained this compound in similar concentrations (*p* > 0.05). Among cereal malts, total protein content was lower in rye than in barley (*p* < 0.05). Furthermore, barley malt showed almost two-fold higher α-amylase activity than rye malt, while β-amylase activity in the studied malts was similar (*p* > 0.05).

### 2.2. Chemical Composition of Mashes before and after Fermentation

The chemical composition of the prepared distillery mashes differed depending on the method of pretreatment of unmalted cereals as well as the type of malt used; the obtained results are presented in Table 2.

The extract content of sweet mashes ranged between (164.4 ± 2.5) g/kg and (182.5 ± 1.5) g/kg for mashes prepared by pressureless liberation of starch on a semi-technical scale and between (162.4 ± 3.5) g/kg and (172.4 ± 4.2) g/kg for those prepared under industrial conditions. As regards samples made from unmalted cereal grains by the pressure-thermal treatment, they were characterized by total extract content ranging from (162.4 ± 3.0) g/kg to (178.4 ± 1.5) g/kg for those prepared on a semi-technical scale and from (159.2 ± 4.5) g/kg to (174.1 ± 3.6) g/kg for those prepared on an industrial scale.

Fermentable sugars present in sweet mashes prepared both on a semi-technical scale and under industrial conditions consisted of glucose at concentrations from (7.1 ± 0.1) to (14.6 ± 0.3) g/L and maltose at concentrations from (56.9 ± 0.9) to (77.3 ± 1.6) g/L. Moreover, all mashes contained from (7.5 ± 0.1) to (15.2 ± 0.5) g of maltotriose per L of mash and from (20.4 ± 1.2) to (46.5 ± 2.3) g of dextrins per L of mash. As regards mashes prepared on a semi-technical scale by the PLS method, an increase in the content of cereal malts from 50% to 100% significantly improved the initial degree of starch saccharification (*p* < 0.05), leading to lower dextrins concentrations, especially in mashes prepared from barley malt. However, this was not true for analogous trials conducted under industrial conditions. Initial pressure-thermal treatment of unmalted cereals did not have a major effect on starch saccharification (Table 2).

Chemical analysis of fermented mashes consisted of the determination of apparent and real extract, as well as the concentration of ethanol, reducing sugars (glucose and maltose), maltotriose, and dextrins (see Table 3). After 72 h of fermentation, the lowest values of apparent extract, i.e., (12.7 ± 1.0) to (16.1 ± 0.5) g/L, were found for mashes prepared exclusively from cereal malts, both on a semi-technical scale and under industrial conditions. Samples prepared from unmalted cereal grains and cereal malts (rye and barley) were characterized by higher apparent extract values, up to (37.0 ± 2.5) g/L.

When assessing ethanol concentration (for the semi-technical scale), lower values were found for mashes prepared by the PLS method from unmalted barley and cereal malts than for those made from unmalted rye and malts or from cereal malts only. Initial pressure-thermal treatment of unmalted cereals, especially rye grains, resulted in higher ethanol content in mashes, both in the semi-technical scale process (between 65.5 ± 2.3 and 67.9 ± 2.2 g/L) and in the industrial scale process (between 65.5 ± 2.1 and 68.6 ± 2.0 g/L) in comparison with samples prepared by the PLS method (Table 3).

As regards residual sugar determined in mashes upon fermentation completion, relatively high concentrations of dextrins (7.6 ± 0.5 to 14.5 ± 0.8 g/L) were found in mashes prepared from unmalted cereal grains and cereal malts processed by the PLS method. The application of a pressure-thermal treatment of unmalted cereal grains prior to mashing with cereal malts resulted in a lower content of non-hydrolyzed dextrins remaining in the fermented mashes.

**Figure 1:**
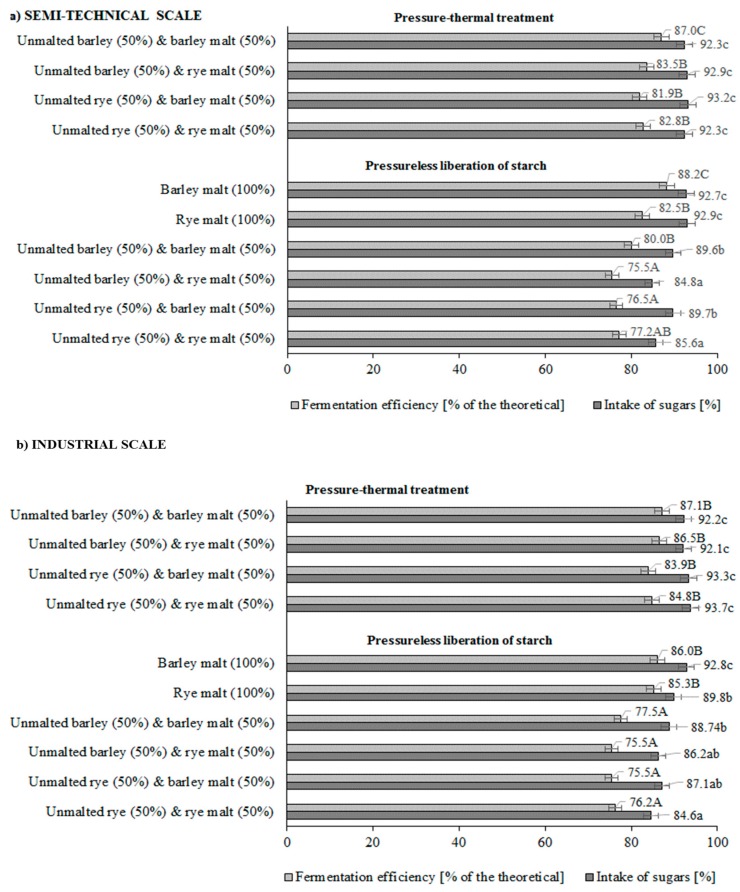
Fermentation factors of distillery mashes prepared from unmalted rye or barley grains and cereal malts. Different letters indicate significant differences (*p* < 0.05) between mean values of intake of sugars (lowercase letters), fermentation efficiency (capital letters).

The degree of sugar utilization and efficiency of ethanol biosynthesis (expressed as percentage of the theoretical amount) were calculated to evaluate fermentation results (Figure 1). A higher sugar consumption rate was found for mashes produced with pressure-thermal treatment of unmalted cereal grains (both on a semi-technical and industrial scale). Fermentation efficiency (actual yield relative to the theoretical amount) for those mashes ranged from 81.9% ± 1.6% to 87% ± 1.7% and was higher (*p* < 0.05) than in samples prepared by the PLS method (75.5% ± 1.5% to 77.5% ± 1.6%). Among the latter mashes, the highest efficiency was revealed by fermented samples prepared from cereal malts only (both on a semi-technical and industrial scale).

### 2.3. Chemical Composition of the Obtained Distillates

A comparison of the chemical composition of the obtained agricultural distillates demonstrates a significant effect of cereal malt, the method of sweet mash preparation, as well as distillation conditions (Table 4 and Table 5).

Among the distillates produced from mashes prepared by the PLS method, the highest concentrations of acetaldehyde (13.51 ± 0.75 mg/L absolute alcohol for the semi-technical scale and 16.05 ± 0.75 mg/L absolute alcohol for the industrial scale) were found in distillates derived from rye malt alone (*p* < 0.05). In turn, among spirits obtained from mashes with pressure-thermal treatment of cereal grains, the highest acetaldehyde content was found in distillates obtained from mashes from unmalted rye grain and cereal malts (12.09 ± 0.58 to 14.26 ± 0.85 mg/L absolute alcohol). All other distillates contained acetaldehyde at lower concentrations (Table 4).

In addition, the presence of ketones, i.e., 2,3-butanedione (1.27 ± 0.38 to 4.78 ± 0.41 mg/L) and aldehydes, such as furfural, was determined in the studied distillates. Significantly lower concentrations of furfural (0.06 ± 0.01 to 3.68 ± 0.32 mg/L absolute alcohol) occurred in distillates produced under industrial conditions, while those obtained on a semi-technical scale contained this compound in amounts between (25.58 ± 1.56) mg/L and (70.05 ± 3.23) mg/L absolute alcohol. All distillates obtained under industrial conditions contained similar concentrations of caprylic aldehyde (20.74 ± 1.15 to 22.58 ± 1.33 mg/L absolute alcohol, *p* > 0.05), irrespective of mash composition and preparation method (Table 3).

In terms of esters, the highest concentrations (*p* < 0.05) were observed for ethyl acetate. No clear effect of the composition of sweet mashes or method of their preparation was noted, with significantly higher concentrations of ethyl acetate found in distillates obtained on a semi-technical scale (144.41 ± 5.50 to 577.72 ± 8.60 mg/L absolute alcohol) compared to those produced under analogous industrial conditions (8.47 ± 0.55 to 67.40 ± 1.55 mg/L absolute alcohol). Moreover, distillates obtained under industrial conditions were characterized by the presence of acetaldehyde diethyl acetal (42.70 ± 1.30 to 129.47 ± 4.20 mg/L absolute alcohol) and ethyl heptanoate (0.02 ± 0.00 to 0.22 ± 0.01 mg/L absolute alcohol), while those compounds were not detected in the spirits obtained on a semi-technical scale.

The tested distillates exhibited small amounts of esters, such as ethyl butyrate, isoamyl acetate, as well as esters of higher carboxylic acids and ethanol, including ethyl caproate, ethyl benzoate, ethyl caprylate, ethyl caprate, and ethyl myristate, as compared to the predominant ethyl acetate.

Among the identified alcohols, methanol concentrations in the obtained distillates (raw spirits) varied widely (*p* < 0.05) between (42.5 ± 6.5) mg/L and (198.1 ± 17.3) mg/L absolute alcohol. Distillates obtained from mashes fermented under industrial conditions exhibited higher concentrations of methyl alcohol as compared to those produced on a semi-technical scale (Table 4).

In the obtained raw spirits, the most abundant higher alcohol was 3-methyl-1-butanol, especially in the distillates produced on a semi-technical scale. In addition, isobutanol, 2-methyl-1-butanol, and 1-propanol were also found at relatively high concentrations. It should be noted that the industrial distillates, and especially those obtained by the PLS method (besides the ones produced exclusively from malts), were characterized by lower concentrations of isobutanol (approximately 12% to 78%) and 2-methyl-1-butanol (approximately 65%) in comparison with spirits obtained on a semi-technical scale. The opposite was true as regards 1-propanol content, which was more than three times higher in some distillates obtained under industrial conditions than in analogous samples of spirits produced on a semi-technical scale. The latter also contained 2-phenylethanol (201.65 ± 12.23 to 282.66 ± 14.20 mg/L absolute alcohol) and trace amounts of benzyl alcohol (0.10 ± 0.01 to 0.65 ± 0.05 mg/L absolute alcohol) (Table 4).

## 3. Discussion

The factors determining the quality of agricultural distillates (also known as raw spirits) obtained from starchy raw materials include: the type and quality of the raw materials, the method of starch liberation (pressureless or pressure-thermal) and saccharification, the type of yeast, as well as fermentation and distillation conditions [11].

Storability is a characteristic criterion of quality, which expresses the ability of grains to maintain their sensory characteristics and technological usefulness without alteration [12]. The basic indicator of the storability of cereal grains, affecting the risk of mold formation and the growth of other undesirable microorganisms is moisture content, which is deemed safe below approximately 15% (wet basis) and 15 °C, as it allows little metabolic activity. Storage of grains with moisture content of more than 16% is not recommended unless steps are taken to prevent mite proliferation, mold growth, and other metabolic activity [13]. In cereal malts, moisture content should usually be kept below 5% [14]. The raw materials used in this study were unmalted rye and barley grains, as well as the corresponding malts, fulfilling the above moisture content requirements, which indicates their high quality.

A significant criterion in selecting raw materials for efficient ethanol production is sugar content. Cereal grains are valued for their high efficiency because they are rich in starch [15]. The starch content of the rye grain used in the study (cv. Dańkowskie Diament) was consistent with the literature data [15]. In turn, the starch content of cereal malts was lower as a result of the development of enzymes hydrolyzing starch to soluble saccharides [16,17]. During mashing, fermentable carbohydrates are produced as a result of enzymatic degradation of starch [18]. The obtained results show a significant difference between the amount of reducing sugars contained in unmalted rye and barley, and in cereal malts.

Protein content is yet another essential determinant of malt quality. High protein content decreases the available carbohydrates, adversely influencing the fermentation process. On the other hand, proteolysis (protease hydrolysis producing amino acids and peptides) during malting and mashing is necessary for yeast metabolism [19]. As regards protein content, it was higher in unmalted cereals than in malts. This was due, amongst others, to the fact that some proteins were used up during the controlled germination of cereals in the malting process [20].

Native starch is composed of essentially linear amylose and branched amylopectin. Both amorphous and crystalline starch elements are found in granule structure, with some minor additions, such as lipids, proteins, and phosphates [21]. Compositional and structural differences may be found not only between starches isolated from different botanical sources, but also between those obtained from the same botanical source [22].

Starches from rye, similarly to those from wheat, triticale, and barley, are composed of large (A-type) and small (B-type) granules with diameters of 23–40 μm and less than 10 μm, respectively. Fractionation of non-granular rye starches resulted in three fractions, i.e., amylose (AM) (22.9%–24.5%), amylopectin (AP) (70.4%–73.2%), and a so-called “intermediate” fraction (IM) (3.2%–6.2%), behaving neither as regular AM nor as regular AP. The average degree of polymerization (DP) for amylose was reported to be in the range of 223–242. In rye starch, there are two populations of amylopectin branch chains, with average DPs of 11–25 and 52–60, respectively [23]. As regards barley starch, large granules constitute 10%–20% of the total number of starch granules and 85%–90% of total starch by weight, while small granules account for 80%–90% of the total by number and 10%–15% by weight. About 25%–55% of amylose in barley starch granules is branched with 4–18 branch points per molecule and a branch chain length of 4 to more than 100. Amylopectin is highly branched and composed of thousands of linear α-1, 4-D glucan unit chains and 4%–5.5% of α-1, 6-glucosidic bonds [24]. The amylose to amylopectin ratio in granules significantly affects the physicochemical and functional properties of starch, such as gelatinization temperature, solubilization, and viscosity of the obtained solutions [25,26].

Based on the above-mentioned literature [21,22,23,24,25,26], it can be inferred that differences in the molecular structure of starch affect the efficiency of its bioconversion to fermentable sugars during ethanol production.

Starch hydrolysis to fermentable carbohydrates (glucose, maltose, and maltotriose) is carried out by malt enzymes, such as α-amylase, β-amylase, limit dextrinase, and α-glucosidase [27]. As enzymatic activity is highly dependent on temperature, the manipulation of such variables is the main control mechanism in the mashing process. In brewing technology, mashing consists of several temperature steps, each favoring different malt enzyme activities. A temperature of 45–50 °C is optimal for the activity of β-glucanases (cell wall degrading enzymes), while proteases are more efficient at 52 °C, β-amylase at 60–65 °C, and α-amylase at 72 °C. The two main starch-digesting enzymes released from malt are α- and ß-amylase. Temperatures of 60–65 °C maximize the activity of β-amylase, while 72 °C is optimal for α-amylase activity [28,29]. A significant reduction in energy consumption and brewing cycle time may be obtained by the application of single-step mashing at 60 °C. [30]. Distillery mashes with the addition of cereal malts (as a source of amylases and starch) were prepared in the experimental part of this work pursuant to the latter solution.

The chemical composition of distillery mashes depends on the type of raw materials and methods of their preparation. According to Hübner et al. [31], extract content in rye malts is significantly higher than in barley malts because rye does not have husks, which represent about 10% of barley dry weight. The obtained results are in agreement with the findings of the above-mentioned authors.

Measurement of the concentrations of fermentable sugars and dextrins in the tested sweet mashes revealed that those prepared from unmalted barley grains and rye malt (exhibiting relatively low α-amylase activity) had the highest content of non-hydrolyzed dextrins, especially when the PLS method was applied. This may probably be attributable to the higher content of amylopectin in barley starch as compared to rye starch, as well as to the higher degree of its polymerization [24].

In Scotch whisky production, malted barley is used as a source of both α-amylase and the exoenzyme β-amylase. The major fermentable sugar produced is maltose, a dimer of two glucose units. Glucose and maltotriose are also present, albeit at lower concentrations [32]. Moreover, limit dextrins are formed in the mashes as a result of amylolytic degradation of amylopectin and are further hydrolyzed by limit dextrinases, which was corroborated by the results obtained in the presented study. Maltose and maltotriose pass intact through the cell membrane by an active process, and, once inside, they are hydrolyzed to glucose units by the α-glucosidase system. In batch fermentation, the fermentation of maltotriose tends to begin later than that of glucose and maltose.

It should be noted that cereal malts are not boiled, so all microorganisms that can survive at the mashing temperature will continue to be active during fermentation. Undesirable microorganisms competing with yeast for nutritional substances impede fermentation and slow it down [33]. Therefore, in this study, a hop α-acid preparation was added to the mashes prior to fermentation as an inhibitor of microbial infections [34].

In the context of enabling high yeast activity during fermentation, free amino nitrogen (FAN) is especially important for yeast growth at the beginning of the fermentation process [35], as a deficiency of vital nutrients may lead to stuck fermentation [36]. Considering the above-mentioned aspects, to ensure appropriate conditions for yeast fermentation activity and prevent the development of bacterial infections, before yeast inoculation the studied distillery mashes were supplemented with the ammonium ion, supplied as a phosphate salt (NH_4_)_2_HPO_4_.

During any fermentation, yeast secretes H+ ions causing a pH decline in the medium. For example, an all malt wort pitched with a pure culture of distiller’s yeast will have an initial pH of approximately 5.2–5.5, which will fall to pH 4.0–4.5 (depending on the solids), and then increase slightly during the stationary phase due to the release of amino acids from autolyzing yeast cells. In fermentation showing late growth of lactic acid bacteria (LAB), these bacteria can use the yeast autolyzate as a nutrient and further lower the pH to approximately 3.8. Contamination by LAB early in fermentation is undesirable and results in a reduced spirit yield since the sugars used up by the bacteria are no longer available to the yeast for ethanol production. The pH value of the prepared sweet mashes was set at 4.8 (acidified from 5.6–5.8), while after process completion it decreased to 4.2–4.3 (data not shown), which is consistent with literature data and confirms the correct duration of fermentation [37].

The indicator used in distilleries for assessing the degree of fermentation is apparent extract, measured in the presence of ethanol. In the case of well-fermented distillery mashes with an initial extract of approximately 180 g/kg, apparent extract should not exceed 10–15 g/kg [38]. During whisky production, the fermentation process is usually allowed to proceed to a point at which the specific gravity of the fermented mash drops to below 1.0 [32]. In the presented study, the lowest values of apparent extract were found in mashes prepared exclusively from cereal malts, both on a semi-technical scale and under industrial conditions. On the other hand, mashes prepared from unmalted grains and cereal malts were characterized by higher apparent extract values, reaching (37.0 ± 2.5) g/L, which could be attributable to the fact that cereal grains contain various amounts of non-starch polysaccharides (NSPs) that are composed predominantly of arabinoxylans (pentosans), β-glucans and cellulose [39]. The detrimental influence of soluble NSPs is mainly associated with their viscosity and physiological effects on the digestive medium. The content and type of NSPs differ among cereals. The amount of NSP relative to dry matter is lower in wheat (11.4%) than in rye kernels (13.2%). Arabinoxylans (AXs) are the predominant NSPs in wheat (6%–8%) and rye (8.9%), while β-glucans are prevalent in barley (7.6%) [40]. Most AXs found in cereal grains are insoluble in water, but those not bound to the cell walls, which can form highly viscous solutions and absorb an amount of water equivalent to about ten times their weight, are known as water extractable AXs (WEAXs). These compounds are important for the purposes of brewing and agricultural distillate production. Soluble NSPs increase medium viscosity, generally hampering the digestion process, whereas insoluble NSPs impede the access of endogenous enzymes to their substrates by physical entrapment [31].

A decrease in dextrin content during fermentation can be attributed to the continuous and simultaneous action of α-amylase, β-amylase, and limit dextrinase. The activity of α-amylase results in an increase in (shorter) dextrins while β-amylase removes a maltose moiety from the non-reducing end of all dextrins. If the combined activity of malt α- and β-amylases were sufficiently random, this could be expected to result in a general reduction in dextrin concentration [41].

As regards the non-hydrolyzed dextrins remaining in the tested mashes upon fermentation completion, relatively higher concentrations of those compounds were found in mashes prepared from unmalted cereal grains and cereal malts by the PLS method than in trials in which unmalted grains were subjected to pressure-thermal treatment in a Henze steamer. Under a water vapor pressure of 0.4 MPa (150 °C), the cellular structure of cereal grain was destroyed, enabling the release and accessibility of starch to enzymes [15]. Moreover, the relatively low β-amylase activity of the applied cereal malts is the probable cause of incomplete starch hydrolysis.

In order to evaluate fermentation results, the degree of sugar utilization and the efficiency of ethanol biosynthesis (expressed as percentage of the theoretical amount) were determined. In this work, the degree of sugars intake was calculated from the difference in the content of total fermentable sugars in mashes before and after the fermentation process, whereas the yield of ethanol production was calculated according to the stoichiometric equation of Gay–Lussac. When comparing the fermentation factors which depend on the method of mash preparation (i.e., pressureless or pressure-thermal pretreatment of unmalted cereals), higher values of sugar consumption and ethanol yield were found for mashes from rye and barley grains pretreated by steaming. The pretreatment of unmalted raw materials, i.e., rye and barley grains, was carried out in a Henze steamer at 0.4 MPa and 151 °C for 45 min. The purpose of this pretreatment was to gelatinize and liquefy starch before its saccharification to fermentable sugars [42]. An important step in the process is mass extrusion. At the end of barothermal treatment, a drain valve in the steamer is opened, and liquid starch mass is rapidly transferred from the steamer, where the pressure is maintained at 0.4–0.5 MPa, to the mash tun, where atmospheric pressure prevails [43]. At this stage of process, the cellular structure of rye grain is destroyed, enabling the release of starch and increasing its accessibility to enzymes.

Among the studied mashes prepared by the PLS method, higher values of sugar intake and fermentation efficiency were observed in trials prepared exclusively from cereal malts than in the ones in which half the weight of raw material used for preparation of sweet mashes consisted of unmalted cereals. At higher malt dosages, malt provides a rich source of α-amylase, which has a greater effect than β-amylase, thus resulting in lower levels of maltose and higher levels of glucose and maltotriose. While it is clear that the addition of malt leads to higher levels of soluble sugars and the addition of unmalted cereals results in a certain carbohydrate levels, it is also important to consider the hydrolytic effects which the endogenous malt starch-degrading enzymes bring into the system [44]. Moreover, fermentation efficiency (especially on a semi-technical scale) was significantly higher in the mash made with barley malt than in that made with rye malt, probably due to the almost two-fold higher activity of α-amylase, faster release of fermentable sugars, and their utilization by yeast.

During the fermentation process, yeast produces ethanol and carbon dioxide, which promote the synthesis of alcohols, esters, and organic acids, and thus determine the flavor and aroma of alcoholic beverages [45]. Evaluation of the chemical composition of the obtained agricultural distillates showed a significant effect of cereal malts, the method of sweet mash preparation, as well as distillation conditions.

Aldehydes and ketones, known as carbonyl compounds, are intermediates in two-step decarboxylation of alpha-keto acids to alcohols as well as in the synthesis and oxidation of alcohols. These volatiles are often noted to have a negative influence on the quality of spirits. The concentrations of carbonyl compounds in agricultural distillates depend on the quality of raw materials, their chemical composition, the conditions of technological processes, and microbial contamination [46]. Inappropriate hygienic and technical parameters lead to increased levels of aldehydes and other by-products in the distillates [15]. The concentration of acetaldehyde in the distillates obtained in the present study was very low (several mg/L) and fulfilled the requirements concerning acetaldehyde content in agricultural distillates (<100 mg/L absolute alcohol) [47]. This confirms the correct duration of mashing and fermentation, as well as the absence of microbial infections as a result of the use of a preparation of hop α-acids [34].

One of the heterocyclic aldehydes occurring in agricultural distillates is furfural, mainly formed during the dehydration of pentoses in technological processes carried out at elevated temperatures [48]. Furfural arises during distillation involving the Maillard reactions [49], so its synthesis in the heated pot still was probably a fundamental factor causing its constant increase. In the tested distillates, significantly higher concentrations of furfural were present in the spirits distilled by using a copper alembic with a column (fermentation on a semi-technical scale) than in the distillates obtained from a 2-column continuous apparatus in the agricultural distillery.

Other ubiquitous compounds in alcoholic beverages include diketone 2,3-butanedione (diacetyl) with a buttery aroma, as well as acetals, which are rapidly formed in distillates. The most prominent of the latter group is acetaldehyde diethyl acetal (1,1-diethoxyethane), with the highest levels among whiskies found in malt whisky [50]. All the tested distillates contained 2,3-butanedione at concentrations from (1.27 ± 0.008) to (4.78 ± 0.40) mg/L absolute alcohol (no correlation between the type of raw material, method of mash preparation, and distillation conditions was observed). On the other hand, acetaldehyde diethyl acetal was only detected in the distillates produced on an industrial scale, which suggests a significant effect of distillation on its formation.

An important group of flavor compounds found in spirits consists of esters (mostly ethyl esters of monocarboxylic acids). Ethyl acetate is quantitatively the most important component of the ester fraction, usually accounting for over 50% of the total. Many short-chain esters, such as isobutyl acetate, ethyl 3-methylbutyrate, ethyl *n*-butyrate, 2-methylbutyl acetate, and 3-methylbutyl acetate, have fairly strong odors [51]. In whisky, the concentration of long-chain carboxylic acid esters increases from ethyl hexanoate up to ethyl decanoate and then declines, with C18 ethyl esters typically being the longest esters to be detected [51,52]. In the analyzed distillates, the predominant ester was ethyl acetate. The distillates also exhibited small amounts of isoamyl acetate and esters of higher carboxylic acids and ethanol, i.e., ethyl caproate (ethyl hexanoate), ethyl caprylate (ethyl octanoate), ethyl caprate (ethyl decanoate), and ethyl myristate. It is noteworthy that the observed significant differences in the concentration of those esters were not associated with the composition of mashes or the method of their preparation, as they occurred in similarly prepared samples distilled in an alembic with a column (on a semi-technical scale) and in an industrial 2-column continuous apparatus.

One of the undesirable compounds in spirit distillates is methanol, which is generated through hydrolysis of methylated pectins present in plants and fruits. While methanol does not directly affect the flavor of the distillate, it is subjected to restrictive controls owing to its high toxicity [53]. Methanol concentrations for the different Scotch whiskies (single malt, single grain, blended) range between 4.7 and 16.4 g/hL absolute alcohol (i.e., between 47 and 164 mg/L) [54], while the tested distillates contained between (42.5 ± 6.5) and (198.71 ± 17.3) mg of methanol per liter of absolute alcohol. While EU Regulation no. 110/2008 [5] defines acceptable concentrations of methanol in ethyl alcohols of agricultural origin, wine spirits, and fruit spirits, it does not set any limits on the content of this compound in distillates of agricultural origin. It should, however, be noted that all the obtained distillates meet the requirements of the regulation, which stipulates that the maximum methanol content in ethyl alcohol (rectified spirit) of agricultural origin shall amount to 30 g/hL absolute alcohol (i.e., 300 mg/L).

From a quantitative point of view, an important group of fermentation by-products consists of higher alcohols, represented mainly by n-propanol, and amyl alcohol (with its isomers, i.e., 2-methyl-1-butanol and 3-methyl-1-butanol) [37]. These compounds play an important role in the formation of flavor qualities in spirits, including whisky and others. Malt Scotch whiskies are rich in higher alcohols, with contents often well over 2 g/L [32]. According to the recommendations of the Polish Standard [47], the maximum concentration of those compounds in agricultural distillates used for Starka production is 5 g/L absolute alcohol.

Aylott and MacKenzie [54] carried out research on the authenticity of Scotch whisky. Blended Scotch whiskies, being combinations of many different malts and grains, are diverse and show representative analytical profiles of their constituent parts. The concentrations of 2- and 3-methyl butanol in grain Scotch whisky were relatively low compared to n-propanol and isobutanol. Malt Scotch whiskies were rich in these higher alcohols, with the average 2- and 3-methyl-1-butanol concentration being 190 g/100 L of absolute alcohol, compared to only 30 g/100 L of absolute alcohol in grain Scotch whiskies. In the obtained distillates, the most abundant higher alcohol was 3-methyl-1-butanol. Relatively high concentrations of isobutanol, 2-methyl-1-butanol and 1-propanol were also found. It should be noted that distillates obtained under industrial conditions were characterized by lower concentrations of isobutanol and 2-methyl-1-butanol than spirits obtained on a semi-technical scale. The opposite was noted as regards 1-propanol content, which was over 3 times higher in some distillates obtained under industrial conditions than in analogous samples of spirits produced on a semi-technical scale. The latter distillates also contained 2-phenylethanol and trace amounts of benzyl alcohol. These results indicate that although the content of higher alcohols is strongly associated with the kind of raw material and yeast used for fermentation [11], the type of apparatus used for distillation and the process parameters can modify the content of these by-products.

## 4. Materials and Methods

### 4.1. Raw Materials

Rye grain of the cultivar Dańkowskie Diament and barley grain of the cultivar Karakan (“DANKO” Plant Breeding Ltd., Choryń, Poland) were used as the main starch raw materials.

The source of amylolytic enzymes and also of starch was cereal malts, i.e. rye and barley malt (Munich malt type II), (Strzegom, Poland).

### 4.2. Production of Sweet Mashe

Distillery mashes were prepared by the two following methods.

#### 4.2.1. Pressureless Liberation of Starch (PLS)

Milled rye or barley grain was mixed with milled rye or barley malt at a ratio of 1:1, mixed with water heated to 40 °C (at a ratio of 3.5 L water per 1 kg of mixture of raw materials), and continually stirred and heated to 58–60 °C [55]. The mixture was kept at this temperature for 30 min. The obtained sweet (unfermented) mash was cooled to 30 °C (optimal for yeast inoculation), acidified with a sulfuric acid solution (250 g/kg) from pH 5.6–5.8 to 4.8 (optimal for yeast activity), and supplemented with an aqueous solution of (NH_4_)_2_HPO_4_ (0.2 g/L mash) as a nutrient for yeast. The hop acid preparation IsoStab^®^ (BetaTec GmbH, Germany) was added to the mashes in the amount of 80 mg/L, as an inhibitor of microbial infections.

#### 4.2.2. Pressure-Thermal Pretreatment of Unmalted Cereals

The pretreatment of unmalted raw materials, i.e., rye and barley grains, was carried out in a Henze steamer at 0.4 MPa, 151 °C for 45 min [56]. After the completion of this treatment, the liquid starch mass was rapidly transferred from the steamer to the mash tun to perform starch saccharification, known as mashing. For this purpose, the extruded rye or barley starch mass was cooled in a mash tun to approximately 65 °C, and the obtained liquid mass was blended with a medium prepared from milled malt and water (at a ratio of 1 kg of malt and 1 L of water). The mixture was kept at 60 °C for 30 min. The next steps of mash preparation for fermentation were the same as in the case of the pressureless method described above.

The experiments were carried out on a semi-technical scale in the mini distillery of the Institute of Fermentation Technology and Microbiology (Department of Spirit and Yeast Technology), (Lodz, Poland) and verified on an industrial scale in a Polish distillery (PPHU Zbig-Rol, Prusinowice, Poland).

### 4.3. Yeast Preparation

Fermentation was carried out using the dry distillery yeast Ethanol Red (*S. cerevisiae*) (Fermentis, Division S.I. Lesaffre, France). Prior to the inoculation of mashes, the yeast was hydrated and disinfected (15 min incubation of cells suspended in sulfuric acid solution, pH 2.5, at ambient temperature). The yeast slurry was added to the mashes at a ratio of 0.3 g d.m./L mash. The inoculated mashes were thoroughly mixed prior to fermentation.

### 4.4. Fermentation of Mashes

Alcoholic fermentation of mashes on a semi-technical scale was carried out in 50 L containers, each with 35 L of inoculated mash. The vessels were sealed with air-tight covers, and kept in a thermostated room at 37 ± 1 °C for 72 h. The fermentation of prepared mashes on an industrial scale was carried out in 36,000 L fermentation tanks equipped with a thermometer to control the temperature of the fermenting medium (37 ± 1 °C); the time of fermentation under industrial conditions was also 72 h. Following fermentation, samples were taken for quantification of ethanol concentration, apparent extract (extract of mash containing ethanol), real extract (after ethanol distillation), reducing sugars, and dextrins.

### 4.5. Distillation

The distillation of alcohol from mashes prepared on a semi-technical scale in the mini-distillery was carried out using a copper alembic consisting of three parts: a 50 L pot, an 80 cm long column with a diameter of 15 cm, filled with cooper chips, and a dephlegmator. Under industrial conditions, the distillation was conducted in a 2-column continuous apparatus. In all the obtained agricultural distillates, the ASV was 90% ± 2%.

### 4.6. Analytical Method

Unmalted rye and cereal malts were analyzed for moisture content [57], reducing sugars [58], and starch [59].

The enzyme activities of cereal malts were measured using Megazyme kits (Megazyme Ltd, Bray, Ireland) according to the ICC standard methods [60]. The activities of α-amylase and β-amylase were measured using the Ceralpha method (K-CERA 01/12, kit) and the Betamyl-3 method (K-BETA3 10/10, kit), respectively. Analyses of enzymatic activity were done in triplicate.

One Ceralpha Unit of α-amylase activity is defined as the amount of enzyme required to release one micromole of p-nitrophenol from non-reducing-end blocked p-nitrophenyl maltoheptaoside (BPNPG7) in in the presence of excess thermostable α-glucosidase during one minute at 40 °C [61].

One Betamyl-3^®^ Unit of β-amylase activity is defined as the amount of enzyme required to release one micromole of p-nitrophenol from *p*-nitrophenyl-β-d-maltotrioside (PNPβ-G3) in the presence of excess thermostable β-glucosidase during one minute at 40 °C [62].

Distillery mashes were analyzed for parameters relevant to quality assessment. Total extract, i.e., the concentration of dissolved solids (mostly sugar) in sweet mashes was measured using an areometer with a scale in g/kg [57]. Fermentable sugars, i.e., glucose and maltose, as well as maltotriose were determined by HPLC (Agilent 1260 Infinity, , Santa Clara, CA, USA) with a 7.7 × 300 mm, 8 µm Hi-Plex H column (Agilent Technologies), equipped with a refractive index detector (RID) at 55 °C. Column temperature was maintained at 60 °C and 5 mM H_2_SO_4_ was used as a mobile phase at a flow rate of 0.7 mL/min with a sample volume of 20 µL. Prior to analysis, samples of mashes were mixed with ZnSO_4_ to final concentrations of 10% to induce protein precipitation. The solid debris was removed by filtration through 0.45 µm polyethersulfone (PES) membranes.

Dextrin content was calculated as the difference between total sugars (expressed as glucose) determined after acid hydrolysis and reducing sugars, using a conversion coefficient into dextrins (0.9) and expressed in g/L mash.

Upon fermentation completion, filtered mashes were analyzed for apparent extract (extract of mash containing ethanol) and real extract (after ethanol distillation), both measured using the same method as that for total extract determination and expressed in g/kg mash. Furthermore, ethanol concentration was determined using an areometer with a scale in percent of ethanol by volume [57], and then converted into g/L of mash. In addition, the concentration of residual reducing sugars, maltotriose, and dextrins was assayed.

Chromatographic analysis of volatile compounds in the obtained distillates was carried out using a GC apparatus (Agilent 7890A) with a mass spectrometer (Agilent MSD 5975C) under the conditions described in the paper by Balcerek et al. [63].

### 4.7. Evaluation of Fermentation Results

The intake of sugars (the percentage of sugar consumption during fermentation) was calculated as the ratio of sugars utilized during fermentation to their initial content in the mash, and expressed as a percentage.

Fermentation efficiency was calculated according to the stoichiometric Gay–Lussac equation in relation to total sugars and expressed as a percentage (%) of the theoretical yield [64].

### 4.8. Statistical Analysis

All fermentation variants were prepared and analyzed in triplicate. The results were tested statistically by analysis of variance (ANOVA) at a significance level *p* ≤ 0.05 using Origin 7.5 software (OriginLab Corporation, Northampton, MA, USA).

## 5. Conclusions

Summing up, the obtained results indicate that all the tested types of Polish malts may be successfully used in the manufacturing of agricultural distillates. Taking into account the increased focus on the craft niche and organic (ecological) products, cereal malts can serve as valuable raw materials for micro-distilleries, ensuring appropriate starch saccharification and leading to unique distillates and spirit beverages with an attractive flavor profile.

## Figures and Tables

**Table 1:** Chemical composition of raw materials.

	Raw Material	Unmalted Rye (cv. Dańkowskie Diament)	Unmalted Barley (cv. Karakan)	Rye Malt	Barley Malt
Components					
Moisture [g/kg]		88.0 ± 6.0b	87.0 ± 5.0b	42.0 ± 2.0a	39.0 ± 2.0a
Starch [g/kg]		621.2 ± 15.2c	485.0 ± 14.5b	410.0 ± 16.3a	382.6 ± 17.5a
Reducing sugars [g/kg]		15.2 ± 1.3a	19.3 ± 1.0b	186.5 ± 15.5c	166.2 ± 12.5c
Protein [g/kg d.m.]		105.5 ± 6.5c	100.5 ± 5.0bc	84.5 ± 3.2a	93.8 ± 4.2b
Amylolytic activities:					
α-amylase [CU/g]		n.d.	n.d.	46.89 ± 2.42a	86.83 ± 2.12b
β-amylase [BU/g]		n.d.	n.d.	2.61 ± 0.56a	2.98 ± 0.58a

Results expressed as mean values ± SE (*n* = 3); values with different letters in the same line are significantly different (*p* < 0.05); n.d.—not detected.

**Table 2:** Chemical composition of sweet mashes prepared from unmalted cereal grains (rye and barley) and corresponding malts.

Fermentation Variant	Semi-Technical Scale					Industrial Scale				
	Total Extract (g/kg mash)	Reducing Sugars (g/L mash)		Maltotriose (g/L mash)	Dextrins (g/L mash)	Total Extract (g/kg mash)	Reducing Sugars (g/L mash)		Maltotriose (g/L mash)	Dextrins (g/L mash)
		Glucose	Maltose				Glucose	Maltose		
**Pressureless Liberation of Starch (PLS)**										
Unmalted rye & rye malt (1:1)	164.4 ± 2.5a	9.5 ± 0.2c	71.2 ± 1.5d	11.1 ± 0.3d	36.5 ± 2.2de	162.4 ± 3.5a	9.7 ± 0.2e	63.2 ± 1.5d	10.2 ± 0.2d	27.7 ± 1.5b
Unmalted rye & barley malt (1:1)	169.2 ± 2.5b				39.4 ± 1.5e	163.4 ± 6.5a	11.8 ± 0.3g	69.3 ± 1.3e	9.5 ± 0.1c	22.3 ± 1.2a
Unmalted barley& rye malt (1:1)	170.8 ± 3.0b	7.1 ± 0.1a	61.9 ± 1.2a	9.7 ± 0.2c	46.5 ± 2.0f	165.3 ± 5.5a	7.0 ± 0.1a	56.9 ± 0.9c	7.6 ± 0.1a	46.5 ± 2.3e
Unmalted barley& barley malt (1:1)	170.6 ± 2.5b	9.7 ± 0.2c	63.4 ± 1.5a	9.9 ± 0.2c	34.5 ± 1.2cd	170.0 ± 3.5b	7.5 ± 0.1b	64.3 ± 1.2d	10.8 ± 0.3e	32.6 ± 1.5cd
Rye malt	182.5 ± 1.5d				27.8 ± 1.5b	172.4 ± 4.2b	10.8 ± 0.2f	71.4 ± 1.7ef	11.9 ± 0.4f	30.7 ± 2.5c
Barley malt	171.5 ± 1.0b	10.6 ± 0.3d	63.7 ± 1.5a	14.3 ± 0.5f	20.4 ± 1.2a	171.8 ± 1.5b	14.6 ± 0.3h	72.5 ± 1.5ef	15.2 ± 0.5g	22.9 ± 1.5a
Pressure-Thermal Treatment *										
Unmalted rye & rye malt (1:1)	166.2 ± 2.5ab	8.5 ± 0.2b	73.12 ± 1.6d	11.8 ± 0.2e	32.9 ± 2.5cd	164.5 ± 2.6a	8.6 ± 0.1c	77.3 ± 1.6g	12.4 ± 0.5f	24.0 ± 2.5ab
Unmalted rye & barley malt (1:1)	163.2 ± 2.2a	9.2 ± 0.4c	71.9 ± 1.6d	14.9 ± 0.4f	31.7 ± .4.2cd	163.1 ± 1.3a	8.7 ± 0.1cd	72.2 ± 1.4ef	15.2 ± 0.5g	30.1 ± 2.2c
Unmalted barley& rye malt (1:1)	162.4 ± 3.0a	8.2 ± 0.3b	66.8 ± 1.3b	9.3 ± 0.2b	31.3 ± 2.5c	159.2 ± 4.5a	9.5 ± 0.2e	54.9 ± 0.8b	10.9 ± 0.3e	35.8 ± 1.5d
Unmalted barley& barley malt (1:1)	178.4 ± 1.5c	7.1 ± 0.2a	67.0 ± 1.5b	7.5 ± 0.1a	25.3 ± 3.3b	174.1 ± 3.6b	9.9 ± 0.1d	64.9 ± 0.8a	10.5 ± 0.2b	33.7 ± 3.0cd

* Pressure-thermal treatment was applied only for unmalted cereals; Results expressed as mean values ± SE (n = 3); mean values in columns with different letters are significantly different (*p* < 0.05).

**Table 3:** Chemical composition of mashes prepared from unmalted cereal grains (rye and barley) and corresponding malts after fermentation.

	Parameters	Semi-Technical Scale							Industrial Scale						
		Extract (g/kg mash)		Ethanol (g/L mash)	Reducing Sugars (g/L mash)		Matrotriose (g/L mash)	Dextrins (g/L mash)	Extract (g/kg mash)		Ethanol (g/L mash)	Reducing Sugars (g/L mash)		Matrotriose (g/L mash)	Dextrins (g/L mash)
Variant of Fermentation		Apparent	Real		Glucose	Maltose			Apparent	Real		Glucose	Maltose		
**Pressureless Liberation of Starch (PLS)**															
Unmalted rye & rye malt (1:1)		30.8 ± 1.5d	60.2 ± 4.5c	61.5 ± 1.6b	0.20 ± 0.02d	1.66 ± 0.05g	1.52 ± 0.03d	14.5 ± 0.8f	35.2 ± 2.5d	60.8 ± 3.5c	52.9 ± 1.2a	0.95 ± 0.05g	1.35 ± 0.05b	2.21 ± 0.05g	11.0 ± 0.5f
Unmalted barley & rye malt (1:1)		33.5 ± 1.0e	59.0 ± 4.0c	58.4 ± 1.8ab	0.11 ± 0.01b	0.87 ± 0.12cd	1.12 ± 0.02a	16.9 ± 1.2g	26.8 ± 1.5b	50.2 ± 1.5b	53.7 ± 1.5ab	0.11 ± 0.01a	3.20 ± 0.12f	2.63 ± 0.08h	7.7 ± 0.2e
Unmalted barley & barley malt (1:1)		17.5 ± 0.5b	47.4 ± 3.5b	58.2 ± 2.2ab	0.15 ± 0.01c	0.30 ± 0.10a	1.31 ± 0.02c	9.5 ± 0.6d	15.0 ± 1.5a	52.0 ± 3.3b	56.0 ± 1.3b	0.23 ± 0.01c	1.30 ± 0.10b	1.80 ± 0.03f	8.3 ± 0.6e
Barley malt		16.1 ± 0.5a	37.4 ± 2.5a	63.1 ± 1.2c	0.20 ± 0.02d	0.89 ± 0.05d	1.32 ± 0.05c	4.9 ± 0.5b	12.7 ± 1.0a	42.2 ± 3.0a	69.4 ± 1.5e	0.10 ± 0.01a	2.30 ± 0.10d	1.05 ± 0.05b	5.4 ± 0.8d
Pressure-Thermal Treatment *															
Unmalted rye & rye malt (1:1)		27.1 ± 1.0c	56.3 ± 2.5c	65.5 ± 2.3cd	0.71 ± 0.03f	1.91 ± 0.05h	1.33 ± 0.02c	5.7 ± 1.0b	32.5 ± 2.5cd	59.0 ± 3.0c	65.5 ± 2.1d	0.32 ± 0.01d	1.50 ± 0.06c	1.42 ± 0.05d	3.3 ± 0.5b
Unmalted rye & barley malt (1:1)		34.7 ± 1.5e	61.1 ± 3.0c	67.9 ± 2.2d	0.15 ± 0.01c	2.74 ± 0.12i	1.11 ± 0.04a	4.4 ± 1.2ab	37.0 ± 2.5d	63.4 ± 5.5c	68.6 ± 2.0de	0.82 ± 0.05f	2.60 ± 0.12e	1.22 ± 0.05c	3.4 ± 0.5b
Unmalted barley & rye malt (1:1)		15.5 ± 0.5a	43.1 ± 1.5b	59.2 ± 1.8ab	0.23 ± 0.01d	1.10 ± 0.05e	1.71 ± 0.05e	3.3 ± 0.9a	24.5 ± 1.5b	47.0 ± 3.5ab	60.8 ± 1.6c	0.70 ± 0.04e	2.60 ± 0.05e	1.53 ± 0.03e	2.9 ± 0.2a
Unmalted barley & barley malt (1:1)		24.5 ± 1.2c	44.4 ± 2.2b	56.4 ± 1.5a	0.12 ± 0.02c	0.43 ± 0.02b	1.12 ± 0.01a	5.4 ± 0.5b	30.1 ± 1.8c	51.0 ± 2.8b	56.8 ± 1.2b	0.15 ± 0.01b	2.60 ± 0.05e	1.52 ± 0.02e	2.6 ± 0.2a

* Pressure-thermal treatment was applied only for unmalted cereals; Results expressed as mean values ± SE (*n* = 3); mean values in columns with different letters are significantly different (*p* < 0.05).

**Table 4:** Concentrations of carbonyl compounds and esters in the obtained agricultural distillates.

Method of Mash Preparation	Composition of Mash		Acet-Aldehyde	2,3-Butane-Dion	Caprylic Aldehyde	Furfural	Ethyl Acetate	Acet-Aldehyde Diethyl Acetal	Ethyl Butyrate	Isoamyl Acetate	Ethyl Caproate	Ethyl Heptanoate	Ethyl Benzoate	Ethyl Caprylate	Ethyl Caprate	Ethyl Myristate
		(mg/L Absolute Alcohol)														
Pressureless liberation of starch (PLS)	Unmalted rye & rye malt (1:1)	ST	4.25 ± 0.25b	3.99 ± 0.35f	n.d.	28.63 ± 1.53g	228.70 ± 5.50m	n.d.	0.39 ± 0.04ef	0.76 ± 0.05d	1.84 ± 0.09c	n.d.	0.15 ± 0.02c	3.32 ± 0.15de	3.33 ± 0.15f	0.64 ± 0.03d
		I	9.83 ± 0.52f	2.62 ± 0.22cd	21.13 ± 1.22a	1.91 ± 0.23d	64.30 ± 1.92f	87.02 ± 3.25b	0.46 ± 0.06efg	1.60 ± 0.11g	5.86 ± 0.22e	n.d.	0.01 ± 0.00a	0.45 ± 0.04b	0.03 ± 0.00c	0.01 ± 0.00a
	Unmalted rye & barley malt (1:1)	ST	4.55 ± 0.35bc	3.98 ± 0.28f	n.d.	38.55 ± 2.12h	181.54 ± 3.12l	n.d.	0.49 ± 0.06fg	1.57 ± 0.15g	1.75 ± 0.07c	n.d.	0.19 ± 0.03c	4.26 ± 0.17f	4.04 ± 0.22gh	1.28 ± 0.07g
		I	9.62 ± 0.82ef	3.25 ± 0.25e	20.74 ± 1.15a	0.13 ± 0.01b	44.15 ± 2.32e	102.69 ± 4.15c	0.40 ± 0.05ef	0.41 ± 0.02b	5.47 ± 0.24e	0.05 ± 0.01b	n.d.	0.43 ± 0.04b	0.04 ± 0.00d	0.01 ± 0.00a
	Unmalted barley & rye malt (1:1)	ST	3.63 ± 0.25a	1.99 ± 0.15b	n.d.	25.58 ± 1.56g	172.51 ± 5.15k	n.d.	0.40 ± 0.05ef	0.68 ± 0.04d	1.46 ± 0.06b	n.d.	0.10 ± 0.02b	4.41 ± 0.15f	5.39 ± 0.26i	1.72 ± 0.12h
		I	3.87 ± 0.23a	1.27 ± 0.38a	22.58 ± 1.33a	1.03 ± 0.23c	8.47 ± 0.55a	111.31 ± 4.55d	0.18 ± 0.02c	1.19 ± 0.07f	7.44 ± 0.35g	0.18 ± 0.02cd	0.01 ± 0.00a	0.22 ± 0.02a	0.01 ± 0.00a	0.02 ± 0.00b
	Unmalted barley & barley malt (1:1)	ST	8.92 ± 0.52e	2.18 ± 0.22bc	n.d.	26.84 ± 1.55g	173.5 ± 3.28k	n.d.	0.51 ± 0.04g	2.06 ± 0.12h	2.14 ± 0.09d	n.d.	0.23 ± 0.02d	6.71 ± 0.32h	5.45 ± 0.25i	1.39 ± 0.11g
		I	10.22 ± 0.47f	3.85 ± 0.21	21.08 ± 1.23a	0.16 ± 0.02b	34.69 ± 1.04d	87.70 ± 3.34b	0.14 ± 0.02b	1.20 ± 0.06f	5.80 ± 0.25e	0.04 ± 0.00b	n.d.	0.23 ± 0.02a	0.01 ± 0.00a	0.01 ± 0.00a
	Rye malt	ST	13.51 ± 0.75h	3.04 ± 0.31de	n.d.	35.79 ± 2.25h	144.41 ± 5.50i	n.d.	0.19 ± 0.02c	0.21 ± 0.01a	0.96 ± 0.05a	n.d.	0.15 ± 0.02c	2.21 ± 0.10c	3.90 ± 0.15gh	0.68 ± 0.03d
		I	16.05 ± 0.72j	3.54 ± 0.22e	21.64 ± 1.34a	0.06 ± 0.01a	41.34 ± 1.24e	107.4 ± 4.25c	0.09 ± 0.01a	1.30 ± 0.07f	6.43 ± 0.31f	0.05 ± 0.01b	0.01 ± 0.00a	0.37 ± 0.05b	0.01 ± 0.00a	0.01 ± 0.00a
	Barley malt	ST	8.67 ± 0.62de	4.48 ± 0.32g	n.d.	49.48 ± 2.56j	93.41 ± 2.80h	n.d.	0.14 ± 0.02b	0.98 ± 0.07e	1.01 ± 0.04a	n.d.	0.29 ± 0.02	3.05 ± 0.16d	2.59 ± 0.15e	0.84 ± 0.04e
		I	10.52 ± 0.65f	3.21 ± 0.22e	21.80 ± 1.20a	3.68 ± 0.32f	15.69 ± 0.47b	81.45 ± 3.89b	0.12 ± 0.01b	1.64 ± 0.06g	6.45 ± 0.28f	0.15 ± 0.02c	0.01 ± 0.00a	0.38 ± 0.05b	0.02 ± 0.00b	0.04 ± 0.01c
Pressure-thermal treatment *	Unmalted rye & rye malt (1:1)	ST	13.50 ± 0.85h	4.78 ± 0.41g	n.d.	36.05 ± 1.45h	187.74 ± 5.63	n.d.	3.46 ± 0.22j	0.40 ± 0.02b	1.09 ± 0.05a	n.d.	0.15 ± 0.02c	3.59 ± 0.12e	5.27 ± 0.31i	1.11 ± 0.05f
		I	13.91 ± 0.72hi	3.71 ± 0.32ef	20.77 ± 1.68a	0.07 ± 0.01a	60.54 ± 1.82f	45.22 ± 1.34a	0.58 ± 0.03g	0.55 ± 0.02c	5.48 ± 0.23e	0.02 ± 0.00a	n.d.	0.19 ± 0.03a	0.01 ± 0.00a	0.01 ± 0.00a
	Unmalted rye & barley malt (1:1)	ST	14.26 ± 0.85hi	4.20 ± 0.28fg	n.d.	70.05 ± 3.23k	162.44 ± 4.87j	n.d.	3.17 ± 0.26	1.69 ± 0.08g	1.70 ± 0.05c	n.d.	0.17 ± 0.02c	4.93 ± 0.22g	5.03 ± 0.32i	1.07 ± 0.04f
		I	12.09 ± 0.58g	2.72 ± 0.22d	21.02 ± 1.23a	0.08 ± 0.01a	67.40 ± 1.55g	42.70 ± 1.30a	0.49 ± 0.03fg	1.18 ± 0.06f	5.67 ± 0.24e	0.02 ± 0.00a	n.d.	0.20 ± 0.01a	0.01 ± 0.00a	0.02 ± 0.00b
	Unmalted barley & rye malt (1:1)	ST	3.83 ± 0.21a	2.99 ± 0.18d	n.d.	35.50 ± 3.15h	423.26 ± 5.50n	n.d.	1.60 ± 0.15i	0.70 ± 0.04d	1.01 ± 0.04a	n.d.	0.16 ± 0.02c	3.53 ± 0.16e	4.24 ± 0.18h	1.29 ± 0.08g
		I	5.32 ± 0.44c	2.74 ± 0.20d	21.15 ± 1.56a	3.09 ± 0.05e	30.77 ± 0.92c	108.9 ± 4.15c	0.39 ± 0.02e	1.49 ± 0.09g	5.90 ± 0.22e	0.16 ± 0.02c	0.01 ± 0.00a	0.23 ± 0.03a	0.05 ± 0.01d	0.04 ± 0.01c
	Unmalted barley & barley malt (1:1)	ST	7.51 ± 0.55d	3.32 ± 0.22e	n.d.	42.77 ± 2.23i	577.72 ± 8.60o	n.d.	0.83 ± 0.08h	1.73 ± 0.06g	1.43 ± 0.06b	n.d.	0.19 ± 0.02c	4.07 ± 0.22f	3.67 ± 0.21fg	1.10 ± 0.06f
		I	7.74 ± 0.48d	3.67 ± 0.32ef	21.58 ± 1.80a	3.47 ± 0.25f	30.57 ± 0.70c	129.47 ± 4.20e	0.28 ± 0.03d	0.87 ± 0.07e	6.40 ± 0.31f	0.22 ± 0.03d	n.d.	0.24 ± 0.03a	0.05 ± 0.01d	0.04 ± 0.01c

* Pressure-thermal treatment was applied only for unmalted cereals; ST—semi-technical scale; I—industrial scale; Results expressed as mean values ± SE (*n* = 3); mean values in columns with different letters are significantly different (*p* < 0.05); n.d.—not detected.

**Table 5:** Concentrations of methanol and higher alcohols in the obtained agricultural distillates.

Method of Mash Preparation	Composition of Mash		Methanol	1-Propanol	Isobutanol	1-Butanol	3-Methyl-1-butanol	2-Methyl-1-butanol	Benzyl Alcohol	2-Phenyl-ethanol
		(mg/L Absolute Alcohol)								
Pressureless liberation of starch (PLS)	Unmalted rye & rye malt (1:1)	ST	72.6 ± 8.5bc	733.98 ± 15.25e	1865.54 ± 20.15l	4.50 ± 0.07d	2220.09 ± 18.35l	1026.02 ± 12.30o	0.65 ± 0.05e	231.59 ± 12.55b
		I	83.3 ± 9.2c	2057.9 ± 17.25l	1377.5 ± 15.35j	4.30 ± 0.05d	1904.3 ± 17.56i	363.92 ± 7.65c	n.d.	n.d.
	Unmalted rye & barley malt (1:1)	ST	42.5 ± 6.5a	744.43 ± 14.32e	1564.25 ± 16.89k	9.19 ± 0.06h	1995.51 ± 17.89j	949.35 ± 10.56n	0.31 ± 0.02d	282.66 ± 14.20d
		I	56.8 ± 7.4b	868.3 ± 12.50g	478.60 ± 9.56b	2.22 ± 0.04b	1082.7 ± 15.36b	330.65 ± 5.56b	n.d.	n.d.
	Unmalted barley & rye malt (1:1)	ST	82.1 ± 10.2c	584.11 ± 8.36a	1744.90 ± 19.23	6.86 ± 0.05e	2231.28 ± 18.55l	1070.39 ± 12.54p	0.21 ± 0.02c	282.44 ± 15.20d
		I	190.8 ± 12.6	2140.2 ± 15.68m	1535.23 ± 17.56k	23.4 ± 0.62l	1556.1 ± 12.89f	488.35 ± 6.35d	n.d.	n.d.
	Unmalted barley & barley malt (1:1)	ST	122.6 ± 15.9d	721.91 ± 12.50e	1547.54 ± 18.36k	13.23 ± 0.09j	2334.36 ± 22.25l	1021.69 ± 11.45o	0.14 ± 0.01b	465.08 ± 18.50e
		I	65.9 ± 5.8b	862.6 ± 14.15g	1234.30 ± 14.48gh	3.23 ± 0.05c	917.40 ± 11.50a	289.50 ± 4.32a	n.d.	n.d.
	Rye malt	ST	142.1 ± 5.5e	653.89 ± 11.32c	1163.44 ± 15.52f	4.31 ± 0.03d	1334.75 ± 14.60c	690.73 ± 6.54g	0.14 ± 0.01b	251.69 ± 12.23bc
		I	155.1 ± 12.2e	1028.9 ± 12.35h	2150.90 ± 22.15m	6.90 ± 0.08e	2209.62 ± 24.35l	792.40 ± 8.23k	n.d.	n.d.
	Barley malt	ST	42.9 ± 7.2a	686.40 ± 10.22d	1323.42 ± 14.80i	13.39 ± 0.11j	1908.17 ± 17.63i	802.83 ± 8.15l	0.10 ± 0.01a	269.41 ± 15.35cd
		I	190.3 ± 15.6f	2180.3 ± 14.48n	1468.50 ± 15.63	21.3 ± 0.45k	1473.82 ± 15.32e	912.30 ± 10.12m	n.d.	n.d.
Pressure-thermal treatment *	Unmalted rye & rye malt (1:1)	ST	102.4 ± 12.6cd	737.34 ± 10.89e	1555.58 ± 18.42k	11.09 ± 0.13i	2071.20 ± 18.45k	957.75 ± 10.78n	0.18 ± 0.02c	201.65 ± 12.23a
		I	172.9 ± 10.8	1184.87 ± 12.50i	346.70 ± 8.33a	1.54 ± 0.02a	1450.02 ± 15.60de	538.41 ± 6.45e	n.d.	n.d.
	Unmalted rye & barley malt (1:1)	ST	112.9 ± 10.9d	848.63 ± 10.35g	1142.10 ± 14.78f	24.36 ± 0.52l	1896.04 ± 17.32i	747.78 ± 8.15ij	0.19 ± 0.02c	242.36 ± 15.32b
		I	198.1 ± 17.3f	1965.4 ± 13.32k	631.92 ± 9.25c	2.27 ± 0.03b	1433.44 ± 17.65d	757.69 ± 8.45j	n.d.	n.d.
	Unmalted barley & rye malt (1:1)	ST	142.5 ± 15.5e	617.56 ± 8.58b	1217.09 ± 13.05g	7.83 ± 0.06g	1641.77 ± 18.45g	736.42 ± 7.89i	0.18 ± 0.01c	233.00 ± 15.15b
		I	147.7 ± 12.5e	2088.52 ± 12.50l	771.08 ± 10.63d	4.39 ± 0.05d	2227.89 ± 24.32l	665.96 ± 7.15f	n.d.	n.d.
	Unmalted barley & barley malt (1:1)	ST	83.1 ± 9.7c	801.81 ± 9.63f	1253.28 ± 14.32h	18.26 ± 0.14	1806.62 ± 17.63h	714.21 ± 7.32h	0.14 ± 0.01b	242.45 ± 12.35bc
		I	97.5 ± 8.5cd	1789.58 ± 13.50j	1030.56 ± 12.25e	7.30 ± 0.07f	2525.72 ± 22.65m	1101.14 ± 12.56q	n.d.	n.d.

* Pressure-thermal treatment was applied only for unmalted cereals; ST—semi-technical scale; I—industrial scale; Results expressed as mean values ± SE (*n* = 3); mean values in columns with different letters are significantly different (*p* < 0.05); n.d—not detected.
